# Natural products targeting cellular processes common in Parkinson's disease and multiple sclerosis

**DOI:** 10.3389/fneur.2023.1149963

**Published:** 2023-03-10

**Authors:** Xuxu Xu, Chaowei Han, Pengcheng Wang, Feimeng Zhou

**Affiliations:** ^1^Institute of Surface Analysis and Chemical Biology, University of Jinan, Jinan, Shangdong, China; ^2^Department of Neurology, Shandong Key Laboratory of Rheumatic Disease and Translational Medicine, The First Affiliated Hospital of Shandong First Medical University and Shandong Provincial Qianfoshan Hospital, Shandong Institute of Neuroimmunology, Jinan, Shandong, China

**Keywords:** Parkinson's disease, multiple sclerosis, natural products, neuroinflammation, oxidative stress, cellular process

## Abstract

The hallmarks of Parkinson's disease (PD) include the loss of dopaminergic neurons and formation of Lewy bodies, whereas multiple sclerosis (MS) is an autoimmune disorder with damaged myelin sheaths and axonal loss. Despite their distinct etiologies, mounting evidence in recent years suggests that neuroinflammation, oxidative stress, and infiltration of the blood-brain barrier (BBB) all play crucial roles in both diseases. It is also recognized that therapeutic advances against one neurodegenerative disorder are likely useful in targeting the other. As current drugs in clinical settings exhibit low efficacy and toxic side effects with long-term usages, the use of natural products (NPs) as treatment modalities has attracted growing attention. This mini-review summarizes the applications of natural compounds to targeting diverse cellular processes inherent in PD and MS, with the emphasis placed on their neuroprotective and immune-regulating potentials in cellular and animal models. By reviewing the many similarities between PD and MS and NPs according to their functions, it becomes evident that some NPs studied for one disease are likely repurposable for the other. A review from this perspective can provide insights into the search for and utilization of NPs in treating the similar cellular processes common in major neurodegenerative diseases.

## Introduction

Parkinson's disease (PD), characterized by striatonigral and dopaminergic degeneration and the Lewy body formation, is a major neurodegenerative disorder affecting mainly elderly people (1), while multiple sclerosis (MS) is an autoimmune demyelinating disease of the central nervous system (CNS) and the commonest neurological disabling disease inflicting young adults (2). Historically, PD and MS were considered movement disorders, as the former affects the direct and indirect pathways of basal ganglia that are key to the facilitation of movements, while the latter, with damaged myelin sheaths, axonal loss, and sclera formation, impairing the transmission of action potential and hardening multiple muscles (3, 4). Some other non-movement symptoms are also shared between these two diseases, which include impaired cognition, atrophy, and depression (5).

**Figure 1:**
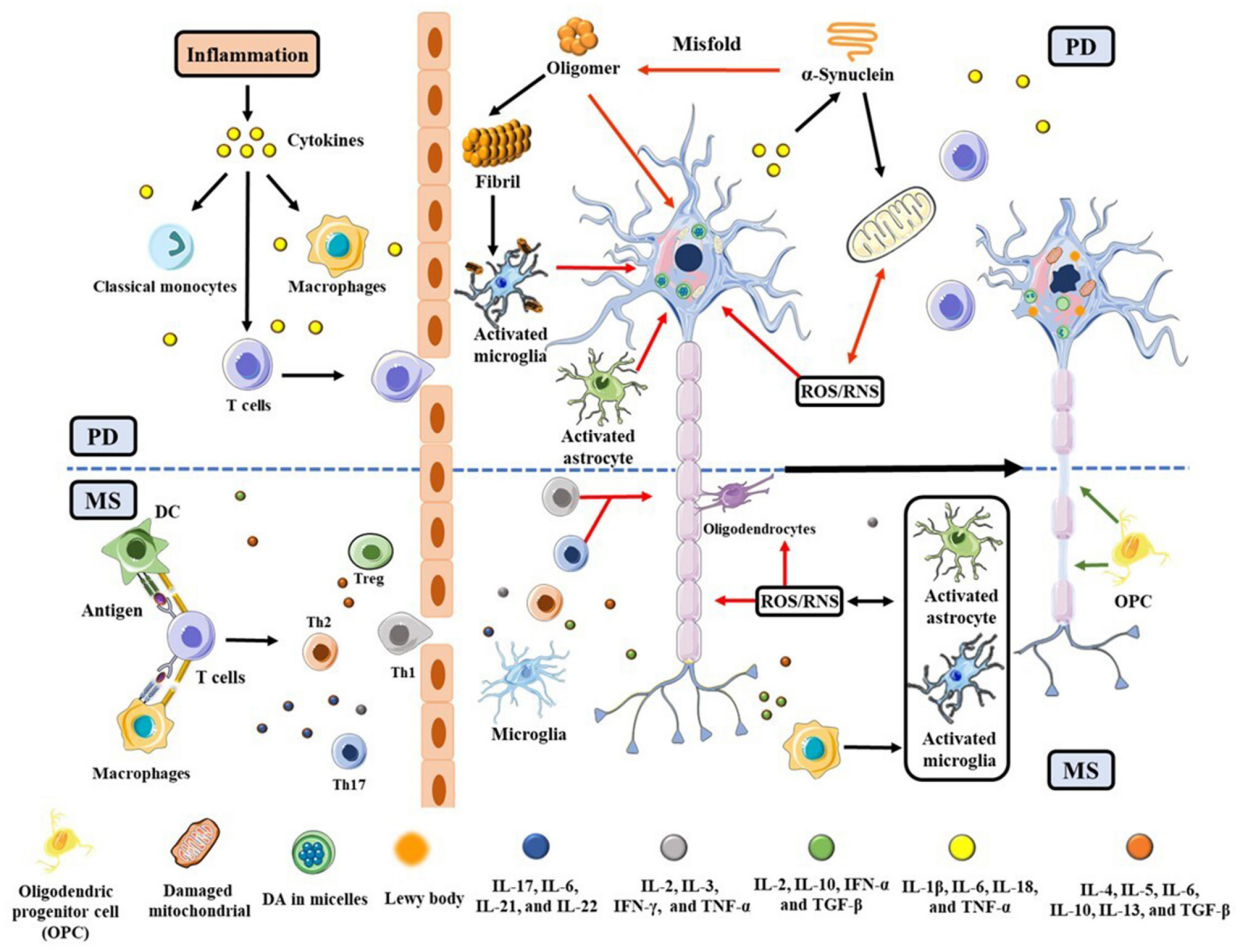
Schematic illustration of PD and MS pathogeneses triggered by neuroinflammation and OS, with an emphasis placed on their commonalities. The red and green arrows represent the neuronal lesions and repairs, respectively. The upper half of the scheme (above the dashed blue line) is related to PD and the lower half is specific to MS.

Although the etiology/pathophysiology of these two diseases appears to be distinctive, mounting evidence suggests that they are caused by exogenous antigens capable of infiltrating toxins or cytokines across a leaky blood-brain barrier (BBB) (6–8). Clinically, patients with MS or other immune disorders were found to have a 33% higher risk of developing PD (9). Genetically, 17 loci on chromosomes are shared by PD and MS (10). Neuroinflammation and oxidative stress (OS) cause cell death, contributing to the ultimate etiologies of PD and MS (11, 12). As shown in Figure 1, T cells, macrophages, and dendritic cells (DCs), along with pro-inflammatory cytokines such as interleukin 6 (IL-6), IL-1β, tumor necrosis factor-α (TNF-α), and reactive oxygen/nitrogen species (ROS/RNS), penetrate the BBB from the peripheral to activate astrocyte and microglial cells. In the presence of cytokines and chemokines, the astrocyte and microglial cells are activated, eliciting a cascade of cellular processes (6, 7). For example, in PD brain α-synuclein (α-syn) becomes misfolded to produce highly neurotoxic oligomers and fibrils (13). The oligomers impact on the integrity of cell membrane, resulting in the death of dopaminergic neurons in the *substantia nigra pars compacta* (SNpc) (14). α-Syn aggregates, which are a major component in the Lewy body, also accumulate at activated microglia (15), further wreaking havoc to neurons. In these dying neurons, damaged mitochondria in turn produce additional ROS to aggravate the situation (16). In MS, neuroinflammation and OS gradually destroy oligodendrocytes, eventually leading to a significant loss of myelin sheaths and the underlying axons in areas as diverse as the brainstem, spinal cord, and optical nerves (17). To offset these damages, cellular defense systems are often stimulated. For example, in MS regulatory T (T_reg_) and Th2 cells secrete anti-inflammatory cytokines such as IL-10 and transforming growth factor (TGF)-β to suppress neuroinflammation (18, 19). In both PD and MS, the Nrf2/Kelch-like ECH-associated protein1 (Keap1) pathway (20) is important for upregulating antioxidative proteins and redox molecules that counteract OS initiated by ROS/RNS.

Thus far, experiments on humans and other primates are limited for both diseases. For PD, disease models are created in animals such as rodents, zebrafish, *Caenorhabditis (C.) elegans*, and *Drosophila* (21). Commonly used neurotoxins are 6-hydroxydopamine (6-OHDA), 1-methyl-4-phenyl-1,2,3,6-tetrahydropyridine (MPTP), paraquat, and rotenone (21). Genetic manipulation of PD-related genes, such as α-syn (SNCA) and protein deglycase (DJ-1), are used in transgenic models (21, 22). PD models induced by inflammatory species such as lipopolysaccharide (LPS) are also employed (23). For MS, the mouse model is used, and the predominant one is the experimental autoimmune encephalomyelitis (EAE), along with cuprizone- and lysolecithin-induced demyelination models (24).

Several cell signaling pathways are related to PD, MS, or both, as listed in Table 1. Nuclear factor-κB (NF-κB) (25), mitogen-activated protein kinase (MAPK) (26), and Janus kinase/signal transducers and activators of transcription (JAK/STAT) (27) all contribute to neuroinflammation. Neuroinflammation is capable of inducing the cell apoptosis and/or pyroptosis pathways such as the nod-like receptor pyrin domain-containing protein 3 (NLRP3)/caspase-1/gasdermin D (GSDMD) (28) and the silent mating type information regulation 2 homolog (SIRT1) (29) pathways in PD and the peroxisome proliferator-activated receptor γ (PPARγ) (30) pathway in MS, which inhibits the NF-κB pathway and stimulates Nrf2 expression to counteract OS. Similarly, the NADPH oxidase pathway also causes OS *via* the production of ROS (31). We should note that these signaling pathways are not independent but interconnected. For instance, NF-κB pathway displays dual effects on OS (32) and Nrf2 can inhibit NF-κB activation (33). Mitochondrial dysfunction is closely linked to PD and other neurodegenerative diseases. Consequently, the peroxisome regulated-activated receptor gamma coactivator-1 alpha (PCG-1α)-NRF-mitochondrial transcription factor A (TFAM) or PCG-1α-NRF-TFAM pathway is generally impacted, leading to impaired oxidative metabolism and mitochondrial biogenesis (34, 35).

**Table 1:** Cell signaling pathways involved in PD and/or MS.

**PD and MS**	**Mainly in PD**	**Mainly in MS**
• NF-κB • MAPK • JAK-STAT • Nrf2 • NADPH oxidase	• SIRT1 • NLRP3/caspase-1/GSDMD • PGC-1α/NRF/TFAM	PPARγ

Because different brain regions are compromised in PD and MS and some cellular processes vary, the clinical modalities are different. Unfortunately, many clinical drugs for PD and MS exhibit limited efficacy and have toxic side effects. One remedy is to resort to the use of natural products (NPs), on the basis that they generally have few side effects. Moreover, many of them are either ingredients in traditional medicines or have been used to treat other neurological disorders, cancers, and diseases related to inflammation (36–38).

Many reviews have summarized the results of using NPs for treating PD, MS, or other neurodegenerative diseases according to their molecular structures (34, 38–44). To our knowledge, few categorized based on their functions toward cellular and subcellar processes inherent in both PD and MS. No reviews have linked the use of NPs for PD to those utilizing the same or similar type of NPs as MS modalities. The motivation behind our attempts to review the NP modalities for both PD and MS stems from the abovementioned similarities and the general belief that therapeutic advances against one neurodegenerative disorder is likely to be useful in targeting the other (45, 46). Both PD and MS have multifactorial traits in their etiology/pathophysiology and molecular mechanisms. Therefore, we focus on those NPs that possess multiple therapeutic effects. Specifically, we emphasize on NPs that are antioxidative/anti-neuroinflammatory, as these properties can help ameliorate both PD and MS (cf. Figure 1). Owing to the limited scope of a mini-review, both NPs and the many cellular and subcellular events reviewed herein are not exhaustive. Furthermore, we only described results that delineated the specific function(s) of each NP and did not include complex mixture in which the role of each species was not elucidated.

## NPs targeting different pathways or cellular processes

**Figure 2:**
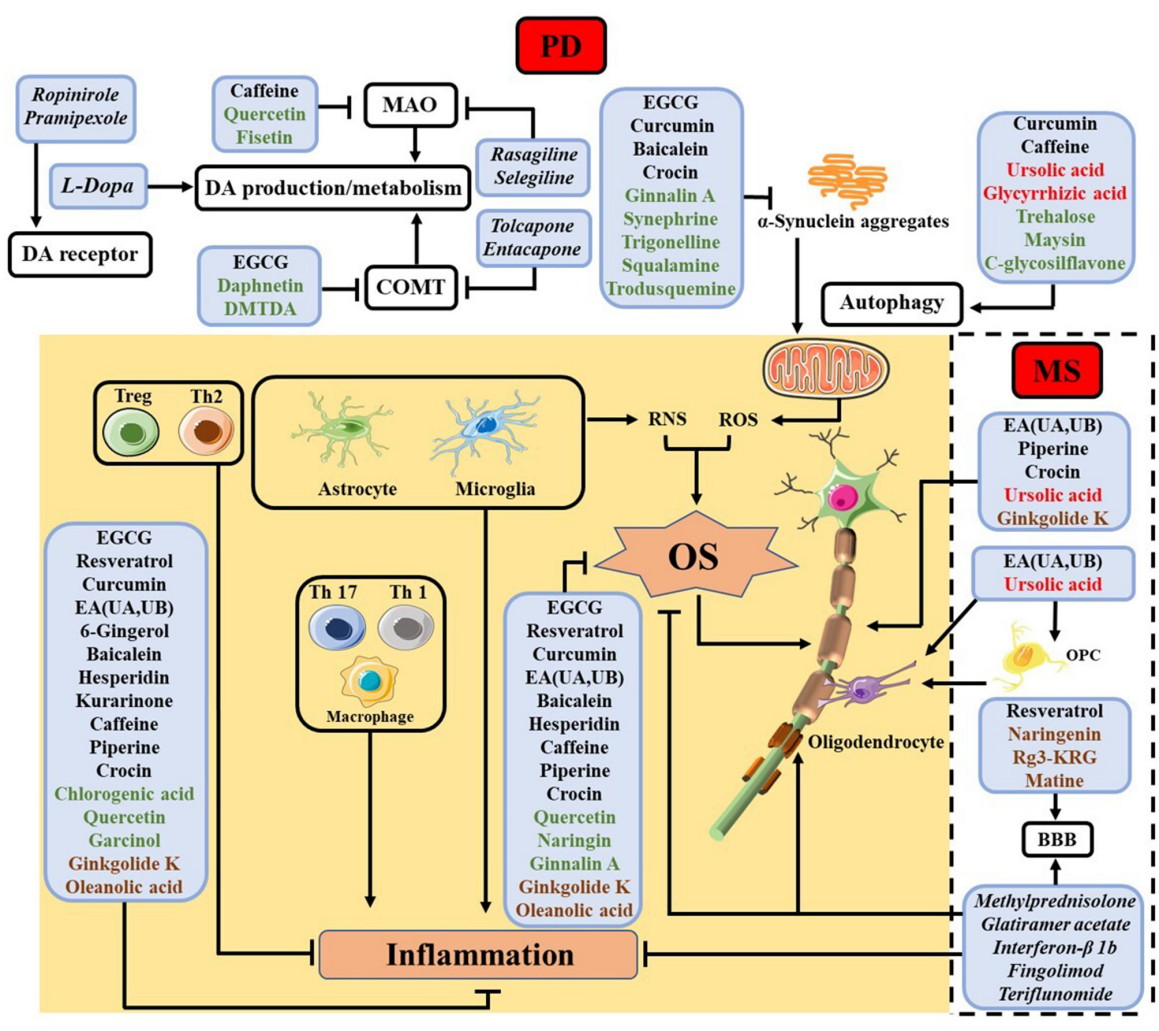
Summary of NPs studied for different cellular processes in PD and MS, with the names of NPs used for the same and different processes in PD and MS shown in black and red, respectively. NPs studied thus far only for PD are shown in green, while those only for MS in brown. The shaded area contains processes related to inflammation and OS common in both diseases, whereas the top unshaded area depicts some extensively studied processes inherent in PD and the unshaded area encircled by the dashed box shows myelin protection and regeneration, which are unique of MS. The names of some clinical drugs targeting different processes are listed in italic. For PD, levodopa (L-Dopa), monoamine oxidase (MAO) inhibitor (rasagiline and selegiline), catechol-*O*-methyl transferase (COMT) inhibitor (tolcapone and entacapone) and DA agonists (ropinirole and pramipexole) are employed (47). In MS, methylprednisolone, glatiramer acetate, interferon-β 1b, fingolimod, and teriflunomide are used (2).

Below we review some findings about the efficacy of select NPs for the various pathways and processes shown in Table 1 and Figure 2, respectively, with emphases placed on the modalities of NPs in counteracting neuroinflammation and OS. We describe the functions and modes of actions of different NPs in the order of names enclosed in the boxes of Figure 2, beginning with those in the shaded area (common in both PD and MS) and progressing to those related only to PD (shown at the top of the figure) and only to MS (encompassed by the dashed box).

### Protection against neuroinflammation

(–)-Epigallocatechin-3-gallate (EGCG), a polyphenol abundant in green tea, can downregulate inducible NO synthase (iNOS) and TNF-α expression, and inhibit neuronal death *via* direct modulation of microglial activation both in SH-SY5Y and in primary rat mesencephalic cultures employed for studying PD (48). It also diminishes IL-6 and IL-1β in LPS-induced rats (48), and reduce the 6-OHDA-induced expression of TNF-α and IL-1β in SK-N-AS cells (49). EGCG also shows anti-inflammatory function toward MS patients and EAE mice (50, 51). The EGCG treatment reverses clinical severity in EAE by modulating the ratio of M1/M2 macrophages both *in vivo* and *in vitro*, with decreased levels of pro-inflammatory cytokines and increased levels of transforming growth factor (TGF)-β (52). Besides, it reduces the levels of phosphorylated NF-κB p65 in M1 macrophages (53).

Resveratrol, another extensively investigated polyphenol, is present in grapes, berries, and peanuts and exerts its anti-neuroinflammatory effect through the NF-κB signaling pathway (54). It suppresses the expression of TNF-α and promotes the expression of IL-10 in BV2 microglia, and mitigates 6-OHDA-induced brain injury (55). Additionally, it renders neuroprotection to MPTP (55) and EAE mice (56).

Curcumin, a polyphenol extract from turmeric, inhibits the secretion of inflammatory cytokines in lipoteichoic acid-induced microglial cells (57) and transforms microglia into the M2-phenotype (58, 59). Additionally, immunomodulatory properties of curcumin were observed in treating MS and EAE (60, 61). Its treatment reduces the clinical severity of EAE by modulating T cell differentiation, with decreased levels of Th1 and Th17-related factors and increased levels of Th2 and T_reg_ responses (18). Besides, it increases the expression of TGF-γ-coding genes in the EAE mice (61).

Other anti-inflammatory phenolic compounds include ellagic acid (EA) and its metabolites such as urolithins A (UA) and B (UB), which decreases the NO level and suppressing expression of cyclooxygenase (COX)-2 and other cytokines in LPS-treated BV2 microglial cells (62). The UA treatment reduces the loss of dopaminergic neurons, ameliorating neuroinflammation in the MPTP mice (63). In addition, EA protects brains of 6-HODA rats (64) and MPTP mice (65) against neuroinflammation. Besides PD, EA has been reported to ameliorate demyelination, reduce MS severity and partially restore tissue levels of TNF-α, IL-6, IL-17A, and IL-10 in EAE rodents (66).

6-Gingerol, a substance in ginger, was found to significantly inhibit 6-OHDA-induced cell apoptosis of PC12 cells through the MAPK pathway (67), and suppresses expressions of TNF-α, IL-6, and iNOS in LPS-induced astrocytes and rats (68). Moreover, in the EAE mouse mode 6-gingerol inhibits the DC activity and Th17 polarization, resulting in induction of tolerogenic DCs (69).

Some flavonoids were found to inhibit the NF-κB pathway. Baicalein, an extract from the plant *Scutellaria baicalensis*, reduces cytokine production in LPS-activated BV2 cells and inhibits the expression of COX-2 and NF-κB/p65 (70). It mitigates the NLRP3/caspase-1/GSDMD pathway in both MPTP (71) and rotenone (72) mouse models. For MS, baicalein alleviates disease severity by reducing Th1 and Th17 cell migration and impairing microglia activation (73), and improves cuprizone-induced EAE mice by inhibiting the ionized calcium binding adapter molecule 1 (Iba1)-positive microglia (74). Furthermore, it decreases levels of CXCR6^+^ CD4^+^, CD8^+^, and Th17 cells in EAE mice (75). Another study showed that it inhibits the M1 macrophage but promotes the M2 macrophage by modulating the STAT1 level (76). Hesperidin, abundant in the citrus fruits, shows efficacy in decreasing many cytokines depicted in Figure 2 in the MPTP mice (77). It also increases the production of IL-10 and TGF-β to confer protection to MS mice (78). Kurarinone, an NP from only in *Sophora flavescens*, is reported to attenuate the MPTP-mediated neuroinflammation (79). In addition, it inhibits clinical progression of EAE by decreasing levels of several pro-inflammatory cytokines and preventing Th1 and Th17 cell differentiation and proliferation (80).

Some alkaloids and triterpenes are also anti-neuroinflammatory. For instance, caffeine suppresses the NF-κB and MAPK pathways in LPS-induced macrophages (81) and attenuates production of cytokines in LPS-induced mouse brain (82) and EAE rats (83). Piperine, an alkaloid in black pepper, depletes pro-inflammatory cytokines in both 6-OHDA rats (84) and EAE mice (85) while enhancing IL-10 production the latter. Glycyrrhizic acid, a triterpene in the licorice plant, decreases COX-2 and iNOS induction in rotenone and MPTP mice (86, 87), and attenuates EAE severity by suppressing pro-inflammatory cytokines (88). Crocin, a major component of saffron, inhibits inflammatory gene expression and ameliorates neuropathology in PD (89) and MS (90, 91).

Other NPs possessing anti-neuroinflammatory properties have only been studied for either PD or MS. Chlorogenic acid, a major component in coffee, inhibits the NF-κB pathway and suppresses IL-1β, IL-6, and TNF-α release in LPS-induced microglia (92). Its supplementation mitigates motor dysfunction in MPTP mice and increases IL-10 (93). Quercetin, present in flowers, leaves, and fruits of many plants, has been shown to suppress inflammatory cytokine levels in LPS-induced primary microglia, zebrafish, and mice, as well as rotenone-induced mice (94). Garcinol, a tri-isoprenylated benzophenone isolated from *Garcinia sp*., is capable of reducing inflammatory markers in the SNpc of MPTP mice (95). Ginkgolide K (96–98) and oleanolic acid (OA) (99) have the same functions in EAE mice by modulating T cell and macrophages/microglia differentiation.

### Protection against OS

EGCG can react with ROS and activate the Nrf2 signaling pathway (48). It inhibits MPP^+^-induced OS in PC12 cells *via* the SIRT1 pathway, and increases glutathione level and mitigates the OS-induced cell death in L-Dopa-treated PC12 cells (48). Moreover, it reduces the NO level and lipid peroxidation in rotenone-induced PD rats (100) and prevents paraquat-induced OS in *Drosophila* (101). In the EAE model, EGCG reduces the ROS level and modulates macrophage subtypes (52). It also directly suppresses M1 macrophage differentiation with lower level of iNOS *in vitro* (52).

Resveratrol was demonstrated to activate the Nrf2 pathway in the brain of rotenone-induced rats (102), and attenuate OS *via* the Nrf2/Keap1 pathway in a microglia cell line (103). Curcumin was found to enhance Nrf2 expression and stability, thereby resisting OS and reducing apoptosis in H_2_O_2_-treated RAW264.7 cells (104). Additionally, it reduces ROS in paraquat-induced SH-SY5Y cells to enhance cell survival (105). EA can inhibit Keap1 to accumulate Nrf2 in the nucleus, which alleviates the impact of ROS on neuronal cells. EA can prevent DA neuron degeneration from OS in MPTP mice (65), reduce ROS level, and reverse the superoxide dismutase and catalase activities in the cuprizone-induced demyelination model (106).

Other compounds also exhibited antioxidant activities. Baicalein (107), hesperidin (108), quercetin (109), caffeine (110), piperine (85), Ginkgolide K (97), and naringin (111) can all activate the Nrf2 pathway, modulate macrophage differentiation, or reduce OS both in cells and in animals. Crocin reduces OS and attenuates damage to dopaminergic neurons in MPTP mice (112). In addition, it inhibits the level of lipid peroxide and increases the total antioxidant capacity in MS patients (91). OA alleviates detrimental effects in EAE mice by reducing lipid peroxidation and superoxide anion accumulation (99). Our group demonstrated that ginnalin A, a polyphenol from the red maple, is a ROS scavenger and can activate Nrf2-regulated antioxidant defense system in SH-SY5Y cells (113).

### Natural products targeting processes inherent in PD and in MS

NPs have also been used as potential therapeutics targeting processes specific to PD or MS, as depicted by the areas at the top of Figure 2 and encompassed by the dashed box, respectively. We briefly review these aspects as follows.

#### Dopaminergic cell preservation and inhibition of protein aggregation

In PD studies, a number of NPs were found to behave similarly to synthetic drugs used clinically, which are shown in italics in Figure 2, to activate DA receptors and inhibit MAO and COMT. EGCG inhibits COMT and preserves the DA level in the brains of LPS-induced rats and MPTP mice (48). Quercetin and fisetin preferentially inhibit MAO-A to MAO-B (114), similar to caffeine (115). Daphnetin, a hydroxycoumarin extract from Daphne species, can halt the COMT-mediated DA *O*-methylation (115). Recently, (1*R*,3*S*)-6,7-dihydroxy-1-methyl-1,2,3,4-tetrahydroisoquinoline-1,3-dicarboxylic acid (DMTDA), a tetrahydroisoquinoline identified in *Mucuna pruriens*, was reported to inhibit COMT (116). It enhances the L-Dopa potency in 6-HODA rats and restores motor behavior of MPP^+^-induced *C. elegans*.

NPs have been identified to inhibit the formation of α-syn oligomers and fibrils, disaggregate aggregates into non-toxic forms, or disrupt their interaction with lipid membranes. EGCG was demonstrated to inhibit fibrillation and disaggregate fibrils, thus enhancing cell survival (48). Curcumin can prevent α-syn aggregation in LPS-induced PD model (117). Baicalein was shown to inhibit fibrillation of the wild-type α-syn and disaggregate fibrils, as one of us found with his co-workers (118). It can also disrupt fibrils of an α-syn mutant (119). Crocin can inhibit α-syn aggregation and disassemble mature fibrils (120). We found that ginnalin A is effective in disrupting the oligomerization and fibrillation of both α-syn and amyloid-β peptides (121), in line with the finding that an NP effective in inhibiting the aggregation of one amyloid species is often capable of acting the same way on another. Alkaloids such as synephrine and trigonelline can also inhibit seed-induced α-syn aggregation, increasing cell viability of SH-SY5Y (122). Squalamine (123) and trodusquemine (124) were reported to inhibit lipid- and fibril-induced α-syn aggregation and alleviate α-syn toxicity to cells. They also showed promising treatment results in animal model studies (124, 125).

Many NPs can modulate autophagy to counteract protein misfolding/aggregation and to attenuate cell death and PD symptoms. Curcumin can enhance autophagy and rescue chloroquine-treated SH-SY5Y cells (105). A chronic caffeine treatment was demonstrated to selectively reverse α-syn-induced autophagy defects in PD mouse striata (126). Ursolic acid, a triterpenoid compound in fruit peels, also promotes autophagic clearance and ameliorates symptoms in rotenone-induced mice (127). Glycyrrhizic acid was reported to alleviate 6-HODA and corticosterone-induced neurotoxicity in SH-SY5Y cells by modulating autophagy (128). Trehalose (129), a disaccharide in some fungi, and maysin (130), the most abundant C-glycosilflavone in corn silk, counteract α-syn toxicity *via* autophagy induction.

#### Preservation and regeneration of myelin

EA promotes oligodendrocyte maturation, decreases oligodendrocyte apoptosis, and decreases demyelination and axonal loss in EAE (66). Additionally, EA, UA, and UB increase the expression of sphingolipids in human oligodendroglioma cells, rendering neuroprotective effects (131). Piperine facilitates myelin repair, suppresses astrocyte activation, and increases the expression of neurotrophins, i.e., brain-derived neurotrophic factor (BDNF) and myelin basic protein (MBP), to attenuate clinical features of the lysolecithin-induced demyelinated model (132). Crocin increases the level of MBP, preserving myelination and axonal density in EAE (90). Ginkgolide K maintains the integrity of myelin and promotes regeneration in EAE *via* the Nrf2 pathway (97).

Ursolic acid, similar to EA, decreases CNS demyelination, preserves axonal integrity, increases the level of ciliary neurotrophic factor (CNTF), and promotes myelin regeneration in a PPARγ-dependent manner (133). Moreover, it enhances myelin repair in EAE mice by promoting the expression of OPC marker transcription factors (134), and alleviates the symptom of cuprizone-induced EAE mice by modulating the IGF-1 expression (135).

#### Protection of BBB

The MS pathogenesis is concomitant with BBB dysfunction, leading to permeation of inflammatory factors across BBB into brain. Resveratrol was found to protect the BBB integrity in EAE mice by suppressing the level of tight junction proteins and inhibiting the expression of adhesion factors ICAM-1 and VCAM-1 (136). Dietary naringenin preserves the BBB integrity in EAE by inhibiting the level of tight junction-associated factors including ZO-1 and occluding (137). Ginsenoside-Rg3-enriched Korean red ginseng extract (Rg3-KRGE) also preserves the BBB integrity, increases the levels of zonula occludens-1, claudin-3, claudin-5, platelet endothelial cell adhesion molecule-1, and fibronectin, and inhibits the level of MMP-9 in EAE by modulating the NADPH oxidase pathway (138). Matrine upregulates the expression of occludin, claudin 5, and tight junction proteins, and attenuates EAE severity (139). These functions are analogous to those of some drugs used currently in clinical settings, which are denoted in italics in the box at the bottom right of Figure 2.

## Conclusions

The diverse functions of the select NPs organized in Figure 2 and reviewed herein bode well with many beliefs in the field while revealing some interesting trends. First, PD and MS share many characteristics, especially in terms of neuroinflammation and OS. It is therefore not surprising that NPs capable of ameliorating PD symptoms have similar effects on MS. In this regard, to select NPs targeting a cellular/subcelluar process of one disease, one can draw on the knowledge of NPs that had been investigated for the same process of the other. A large stockpile of NPs has been examined thus far for only PD or only MS. At least some of them can be repurposed reciprocally or even for other neurodegenerative diseases such as Alzheimer's disease and amyotrophic lateral sclerosis. Second, among the countless NPs, special attention should be given to those that have displayed efficacy in modulating/intervening multiple cellular processes and signaling pathways, owing to the complexity of both PD and MS. Third, from Figure 2 it is apparent that even an NP possessing different functions is incapable of counteracting all the detrimental effects inherent in the many factors or processes. Thus, the combined use of multiple NPs might be needed for regulating the different pathways. Fourth, NPs have shown great promise in addressing the pathological processes for which no clinical drugs are available. Even for processes that have been dealt with by clinical drugs, NPs offer as alternatives to afford equally effective treatments without severe side effects. Finally, an increasingly accepted notion in the PD field is that inflammation is significantly manifested. In particular, increased levels of pro-inflammatory cytokines, activation of the immune cells, and their infiltration through a more permeable BBB are hallmarks being recognized. As these processes have long been studied in the MS field, many NPs and their known functions are likely translatable to PD research and modalities. As the research continues to progress from cellular and rodent models to primates and patients, it is foreseeable that the vast pool of NPs should afford at least a few highly effective therapeutics with low or little toxicity.

## Author contributions

XX, CH, and PW collected materials. FZ formulated the review structure. The first draft of the manuscript was written by XX, CH, and PW. A revision was finalized by FZ and PW. All authors read and approved the final manuscript.

## References

[1] Jankovic J Tan EK. Parkinson's disease: etiopathogenesis and treatment. J Neurol Neurosurg Psychiatry. (2020) 91:795–808. 10.1136/jnnp-2019-322338 32576618

[2] Thompson AJ Baranzini SE Geurts J Hemmer B Ciccarelli O. Multiple sclerosis. Lancet. (2018) 391:1622–36. 10.1016/S0140-6736(18)30481-1 29576504

[3] Bear MF Connors BW Paradiso MA. Neuroscience: Exploring the Brain. Enhanced 4th ed. Burlington, MA: Jones & Bartlett Learning (2020).

[4] Brady ST Siegel GJ Albers RW Price DL. Basic Neurochemistry. Brady ST, Siegel GJ, Albers RW, Price DL, editors. New York, NY: Academic Press (2012). p. 1–1096.

[5] Lai B Young H-J Bickel CS Motl RW Rimmer JH. Current trends in exercise intervention research, technology, and behavioral change strategies for people with disabilities: a scoping review. Am J Phys Med Rehabil. (2017) 96:748–61. 10.1097/PHM.0000000000000743 28398967

[6] Tan E-K Chao Y-X West A Chan L-L Poewe W Jankovic J. Parkinson disease and the immune system - associations, mechanisms and therapeutics. Nat Rev Neurol. (2020) 16:303–18. 10.1038/s41582-020-0344-4 32332985

[7] Tansey MG Wallings RL Houser MC Herrick MK Keating CE Joers V. Inflammation and immune dysfunction in parkinson disease. Nat Rev Immunol. (2022) 22:657–73. 10.1038/s41577-022-00684-6 35246670 PMC8895080

[8] Erkkinen MG Kim M-O Geschwind MD. Clinical neurology and epidemiology of the major neurodegenerative diseases. Cold Spring Harb Perspect Biol. (2018) 10:a033118. 10.1101/cshperspect.a033118 28716886 PMC5880171

[9] Li X Sundquist J Sundquist K. Subsequent risks of parkinson disease in patients with autoimmune and related disorders: a nationwide epidemiological study from Sweden. Neurodegener Dis. (2012) 10:277–84. 10.1159/000333222 22205172

[10] Chang D Nalls MA Hallgrimsdottir IB Hunkapiller J van der Brug M Cai F. A meta-analysis of genome-wide association studies identifies 17 new Parkinson's disease risk loci. Nat Genet. (2017) 49:1511–6. 10.1038/ng.3955 28892059 PMC5812477

[11] Stephenson J Nutma E van der Valk P Amor S. Inflammation in CNS neurodegenerative diseases. Immunology. (2018) 154:204–19. 10.1111/imm.12922 29513402 PMC5980185

[12] Singh A Kukreti R Saso L Kukreti S. Oxidative stress: a key modulator in neurodegenerative diseases. Molecules. (2019) 24:1583. 10.3390/molecules24081583 31013638 PMC6514564

[13] Mehra S Sahay S Maji SK. α-Synuclein misfolding and aggregation: implications in Parkinson's disease pathogenesis. Biochim Biophys Acta Proteins Proteom. (2019) 1867:890–908. 10.1016/j.bbapap.2019.03.001 30853581

[14] Mor DE Tsika E Mazzulli JR Gould NS Kim H Daniels MJ. Dopamine induces soluble α-synuclein oligomers and nigrostriatal degeneration. Nat Neurosci. (2017) 20:1560–8. 10.1038/nn.4641 28920936 PMC5893155

[15] Zhang W Wang T Pei Z Miller DS Wu X Block ML. Aggregated α-synuclein activates microglia: a process leading to disease progression in Parkinson's disease. FASEB J. (2005) 19:533–42. 10.1096/fj.04-2751com 15791003

[16] Dan Dunn J Alvarez LA Zhang X Soldati T. Reactive oxygen species and mitochondria: a nexus of cellular homeostasis. Redox Biol. (2015) 6:472–85. 10.1016/j.redox.2015.09.005 26432659 PMC4596921

[17] Teleanu DM Niculescu AG Lungu II Radu CI Vladâcenco O Roza E. An overview of oxidative stress, neuroinflammation, and neurodegenerative diseases. Int J Mol Sci. (2022) 23:5938. 10.3390/ijms23115938 35682615 PMC9180653

[18] Liu JQ Yan YQ Liu JT Wang YR Wang X. Curcumin prevents experimental autoimmune encephalomyelitis by inhibiting proliferation and effector Cd4^+^T cell activation. Eur Rev Med Pharmacol Sci. (2019) 23:9108–16. 10.26355/eurrev_201910_19314 31696502

[19] Duscha A Gisevius B Hirschberg S Yissachar N Stangl GI Eilers E. Propionic acid shapes the multiple sclerosis disease course by an immunomodulatory mechanism. Cell. (2020) 180:1067–80.e16. 10.1016/j.cell.2020.02.035 32160527

[20] Zgorzynska E Dziedzic B Walczewska A. An overview of the Nrf2/ARE pathway and its role in neurodegenerative diseases. Int J Mol Sci. (2021) 22:9592. 10.3390/ijms22179592 34502501 PMC8431732

[21] Chia SJ Tan E-K Chao Y-X. Historical perspective: models of Parkinson's disease. Int J Mol Sci. (2020) 21:2464. 10.3390/ijms21072464 32252301 PMC7177377

[22] Dawson TM Ko HS Dawson VL. Genetic animal models of Parkinson's disease. Neuron. (2010) 66:646–61. 10.1016/j.neuron.2010.04.034 20547124 PMC2917798

[23] Deng I Corrigan F Zhai G Zhou X-F Bobrovskaya L. Lipopolysaccharide animal models of Parkinson's disease: recent progress and relevance to clinical disease. Brain Behav Immun Health. (2020) 4:100060. 10.1016/j.bbih.2020.100060 34589845 PMC8474547

[24] Smith P. Animal models of multiple sclerosis. Curr Protoc. (2021) 1:e185-e. 10.1002/cpz1.185 34170637

[25] Gasparini C Feldmann M. NF-κB as a target for modulating inflammatory responses. Curr Pharm Des. (2012) 18:5735–45. 10.2174/138161212803530763 22726116

[26] Kim EK Choi E-J. Pathological roles of Mapk signaling pathways in human diseases. Biochim Biophys Acta. (2010) 1802:396–405. 10.1016/j.bbadis.2009.12.009 20079433

[27] Harrison DA. The JAK/STAT pathway. Cold Spring Harb Perspect Biol. (2012) 4:a011205. 10.1101/cshperspect.a011205 22383755 PMC3282412

[28] Li S Sun Y Song M Song Y Fang Y Zhang Q. NLRP3/caspase-1/GSDMD-mediated pyroptosis exerts a crucial role in astrocyte pathological injury in mouse model of depression. JCI Insight. (2021) 6:e146852. 10.1172/jci.insight.146852 34877938 PMC8675200

[29] Hwang JW Yao H Caito S Sundar IK Rahman I. Redox regulation of SIRT1 in inflammation and cellular senescence. Free Radic Biol Med. (2013) 61:95–110. 10.1016/j.freeradbiomed.2013.03.015 23542362 PMC3762912

[30] Cai W Yang T Liu H Han L Zhang K Hu X. Peroxisome proliferator-activated receptor γ (PPARγ): a master gatekeeper in CNS injury and repair. Prog Neurobiol. (2018) 163–164:27–58. 10.1016/j.pneurobio.2017.10.002 29032144 PMC6037317

[31] Belarbi K Cuvelier E Destée A Gressier B Chartier-Harlin MC. NADPH oxidases in Parkinson's disease: a systematic review. Mol Neurodegener. (2017) 12:84. 10.1186/s13024-017-0225-5 29132391 PMC5683583

[32] Lingappan K. NF-κB in oxidative stress. Curr Opin Toxicol. (2018) 7:81–6. 10.1016/j.cotox.2017.11.002 29862377 PMC5978768

[33] Ganesh Yerra V Negi G Sharma SS Kumar A. Potential therapeutic effects of the simultaneous targeting of the Nrf2 and NF-κB pathways in diabetic neuropathy. Redox Biol. (2013) 1:394–7. 10.1016/j.redox.2013.07.005 24024177 PMC3757712

[34] Mohammadipour A. A focus on natural products for preventing and cure of mitochondrial dysfunction in Parkinson's disease. Metab Brain Dis. (2022) 37:889–900. 10.1007/s11011-022-00931-8 35156154

[35] Grünewald A Rygiel KA Hepplewhite PD Morris CM Picard M Turnbull DM. Mitochondrial DNA depletion in respiratory chain-deficient Parkinson disease neurons. Ann Neurol. (2016) 79:366–78. 10.1002/ana.24571 26605748 PMC4819690

[36] Wang Y Chen S Du K Liang C Wang S Owusu Boadi E. Traditional herbal medicine: therapeutic potential in rheumatoid arthritis. J Ethnopharmacol. (2021) 279:114368. 10.1016/j.jep.2021.114368 34197960

[37] Wang S Fu JL Hao HF Jiao YN Li PP Han SY. Metabolic reprogramming by traditional Chinese medicine and its role in effective cancer therapy. Pharmacol Res. (2021) 170:105728. 10.1016/j.phrs.2021.105728 34119622

[38] Chen X Drew J Berney W Lei W. Neuroprotective natural products for Alzheimer's disease. Cells. (2021) 10:1309. 10.3390/cells10061309 34070275 PMC8225186

[39] Pang M Peng R Wang Y Zhu Y Wang P Moussian B. Molecular understanding of the translational models and the therapeutic potential natural products of Parkinson's disease. Biomed Pharmacother. (2022) 155:113718. 10.1016/j.biopha.2022.113718 36152409

[40] Zha Z Liu S Liu Y Li C Wang L. Potential utility of natural products against oxidative stress in animal models of multiple sclerosis. Antioxidants. (2022) 11:1495. 10.3390/antiox11081495 36009214 PMC9404913

[41] Guo Y-X Zhang Y Gao Y-H Deng S-Y Wang L-M Li C-Q. Role of plant-derived natural compounds in experimental autoimmune encephalomyelitis: a review of the treatment potential and development strategy. Front Pharmacol. (2021) 12:639651. 10.3389/fphar.2021.639651 34262447 PMC8273381

[42] Zhang H Bai L He J Zhong L Duan X Ouyang L. Recent advances in discovery and development of natural products as source for anti-Parkinson's disease lead compounds. Eur J Med Chem. (2017) 141:257–72. 10.1016/j.ejmech.2017.09.068 29031072

[43] Yu S Liu M Hu K. Natural products: potential therapeutic agents in multiple sclerosis. Int Immunopharmacol. (2019) 67:87–97. 10.1016/j.intimp.2018.11.036 30537635

[44] Sharifi-Rad M Lankatillake C Dias DA Docea AO Mahomoodally MF Lobine D. Impact of natural compounds on neurodegenerative disorders: from preclinical to pharmacotherapeutics. J Clin Med. (2020) 9:1061. 10.3390/jcm9041061 32276438 PMC7231062

[45] Lamptey RNL Chaulagain B Trivedi R Gothwal A Layek B Singh J. Review of the common neurodegenerative disorders: current therapeutic approaches and the potential role of nanotherapeutics. Int J Mol Sci. (2022) 23:1851. 10.3390/ijms23031851 35163773 PMC8837071

[46] Yacoubian TA. Chapter 1 - neurodegenerative disorders: why do we need new therapies? In:Adejare A, editor. Drug Discovery Approaches for the Treatment of Neurodegenerative Disorders. San Diego, CA: Academic Press (2017). p. 1–16.

[47] Armstrong MJ Okun MS. Diagnosis and treatment of Parkinson disease a review. JAMA. (2020) 323:548–60. 10.1001/jama.2019.22360 32044947

[48] Wang Y Wu S Li Q Lang W Li W Jiang X. Epigallocatechin-3-gallate: a phytochemical as a promising drug candidate for the treatment of Parkinson's disease. Front Pharmacol. (2022) 13:977521. 10.3389/fphar.2022.977521 36172194 PMC9511047

[49] Özduran G Becer E Vatansever HS Yücecan S. Neuroprotective effects of catechins in an experimental Parkinson's disease model and SK-N-AS cells: evaluation of cell viability, anti-inflammatory and anti-apoptotic effects. Neurol Res. (2022) 44:511–23. 10.1080/01616412.2021.2024715 35000557

[50] Afshar B Ganjalikhani-Hakemi M Khalifezadeh Esfahani Z Eskandari N Shaygannajad V Hosseininasab F. Evaluating the effects of epigallocatechin-3-gallate on HIF-1α protein and rorc gene expression in peripheral blood mononuclear cells in patients with multiple sclerosis. Basic Clin Neurosci. (2021) 12:533–40. 10.32598/bcn.2021.2252.1 35154593 PMC8817175

[51] Herges K Millward JM Hentschel N Infante-Duarte C Aktas O Zipp F. Neuroprotective effect of combination therapy of glatiramer acetate and epigallocatechin-3-gallate in neuroinflammation. PLoS ONE. (2011) 6:e25456. 10.1371/journal.pone.0025456 22022398 PMC3192751

[52] Cai F Liu S Lei Y Jin S Guo Z Zhu D. Epigallocatechin-3 gallate regulates macrophage subtypes and immunometabolism to ameliorate experimental autoimmune encephalomyelitis. Cell Immunol. (2021) 368:104421. 10.1016/j.cellimm.2021.104421 34385001

[53] Aktas O Prozorovski T Smorodchenko A Savaskan NE Lauster R Kloetzel P-M. Green tea epigallocatechin-3-gallate mediates T cellular NF-κB inhibition and exerts neuroprotection in autoimmune encephalomyelitis. J Immunol. (2004) 173:5794–800. 10.4049/jimmunol.173.9.5794 15494532

[54] Cianciulli A Dragone T Calvello R Porro C Trotta T Lofrumento DD. Il-10 plays a pivotal role in anti-inflammatory effects of resveratrol in activated microglia cells. Int Immunopharmacol. (2015) 24:369–76. 10.1016/j.intimp.2014.12.035 25576658

[55] Prakash S Carter WG. The neuroprotective effects of cannabis-derived phytocannabinoids and resveratrol in Parkinson's disease: a systematic literature review of pre-clinical studies. Brain Sci. (2021) 11:1573. 10.3390/brainsci11121573 34942876 PMC8699487

[56] Gandy KAO Zhang J Nagarkatti P Nagarkatti M. Resveratrol (3, 5, 4'-trihydroxy-trans-stilbene) attenuates a mouse model of multiple sclerosis by altering the MIR-124/sphingosine kinase 1 axis in encephalitogenic T cells in the brain. J Neuroimmune Pharmacol. (2019) 14:462–77. 10.1007/s11481-019-09842-5 30941623 PMC6900929

[57] Yu Y Shen Q Lai Y Park SY Ou X Lin D. Anti-inflammatory effects of curcumin in microglial cells. Front Pharmacol. (2018) 9:386. 10.3389/fphar.2018.00386 29731715 PMC5922181

[58] Zhang J Zheng Y Luo Y Du Y Zhang X Fu J. Curcumin inhibits Lps-induced neuroinflammation by promoting microglial M2 polarization *via* TREM2/TLR4/NF-Kb pathways in BV2 cells. Mol Immunol. (2019) 116:29–37. 10.1016/j.molimm.2019.09.020 31590042

[59] Qiao P Ma J Wang Y Huang Z Zou Q Cai Z. Curcumin prevents neuroinflammation by inducing microglia to transform into the M2-phenotype *via* CaMKKβ-dependent activation of the AMP-activated protein kinase signal pathway. Curr Alzheimer Res. (2020) 17:735–52. 10.2174/1567205017666201111120919 33176649

[60] Petracca M Quarantelli M Moccia M Vacca G Satelliti B D'Ambrosio G. Prospective study to evaluate efficacy, safety and tolerability of dietary supplement of curcumin (BCM95) in subjects with active relapsing multiple sclerosis treated with subcutaneous interferon B-1a 44 Mcg TIW (CONTAIN): a randomized, controlled trial. Mult Scler Relat Disord. (2021) 56:103274. 10.1016/j.msard.2021.103274 34583214

[61] Esmaeilzadeh E Soleimani M Zare-Abdollahi D Jameie B Khorshid HRK. Curcumin ameliorates experimental autoimmune encephalomyelitis in a C57BL/6 mouse model. Drug Dev Res. (2019) 80:629–36. 10.1002/ddr.21540 31033006

[62] Xu J Yuan C Wang G Luo J Ma H Xu L. Urolithins attenuate LPS-induced neuroinflammation in BV2 microglia *via* MAPK, Akt, and NF-κB signaling pathways. J Agric Food Chem. (2018) 66:571–80. 10.1021/acs.jafc.7b03285 29336147

[63] Qiu J Chen Y Zhuo J Zhang L Liu J Wang B. Urolithin a promotes mitophagy and suppresses NLRP3 inflammasome activation in lipopolysaccharide-induced BV2 microglial cells and MPTP-induced Parkinson's disease model. Neuropharmacology. (2022) 207:108963. 10.1016/j.neuropharm.2022.108963 35065082

[64] Farbood Y Sarkaki A Dolatshahi M Taqhi Mansouri SM Khodadadi A. Ellagic acid protects the brain against 6-hydroxydopamine induced neuroinflammation in a rat model of Parkinson's disease. Basic Clin Neurosci. (2015) 6:83–9. Available online at: http://bcn.iums.ac.ir/article-1-578-en.html 27307952 PMC4636882

[65] Ardah MT Bharathan G Kitada T Haque ME. Ellagic acid prevents dopamine neuron degeneration from oxidative stress and neuroinflammation in MPTP model of Parkinson's disease. Biomolecules. (2020) 10:1519. 10.3390/biom10111519 33172035 PMC7694688

[66] Kiasalari Z Afshin-Majd S Baluchnejadmojarad T Azadi-Ahmadabadi E Esmaeil-Jamaat E Fahanik-Babaei J. Ellagic acid ameliorates neuroinflammation and demyelination in experimental autoimmune encephalomyelitis: involvement of NLRP3 and pyroptosis. J Chem Neuroanat. (2021) 111:101891. 10.1016/j.jchemneu.2020.101891 33217488

[67] Rezazadeh-Shojaee F-S Ramazani E Kasaian J Tayarani-Najaran Z. Protective effects of 6-gingerol on 6-hydroxydopamine-induced apoptosis in PC12 cells through modulation of SAPK/JNK and survivin activation. J Biochem Mol Toxicol. (2022) 36:e22956. 10.1002/jbt.22956 34783140

[68] Zhang F Zhang J-G Yang W Xu P Xiao Y-L Zhang H-T. 6-gingerol attenuates LPS-induced neuroinflammation and cognitive impairment partially *via* suppressing astrocyte overactivation. Biomed Pharmacother. (2018) 107:1523–9. 10.1016/j.biopha.2018.08.136 30257370

[69] Han J-J Li X Ye Z-Q Lu X-Y Yang T Tian J. Treatment with 6-gingerol regulates dendritic cell activity and ameliorates the severity of experimental autoimmune encephalomyelitis. Mol Nutr Food Res. (2019) 63:e1801356. 10.1002/mnfr.201801356 31313461

[70] Yan J-j Du G-h Qin X-m Gao L. Baicalein attenuates the neuroinflammation in LPS-activated BV-2 microglial cells through suppression of pro-inflammatory cytokines, COX2/NF-Kb expressions and regulation of metabolic abnormality. Int Immunopharmacol. (2020) 79:106092. 10.1016/j.intimp.2019.106092 31863920

[71] Rui W Li S Xiao H Xiao M Shi J. Baicalein attenuates neuroinflammation by inhibiting NLRP3/caspase-1/GSDMD pathway in MPTP-induced mice model of Parkinson's disease. Int J Neuropsychopharmacol. (2020) 23:762–73. 10.1093/ijnp/pyaa060 32761175 PMC7745250

[72] Zhao X Kong D Zhou Q Wei G Song J Liang Y. Baicalein alleviates depression-like behavior in rotenone- induced Parkinson's disease model in mice through activating the BDNF/TRKB/CREB pathway. Biomed Pharmacother. (2021) 140:111556. 10.1016/j.biopha.2021.111556 34087694

[73] Xu J Zhang Y Xiao Y Ma S Liu Q Dang S. Inhibition of 12/15-lipoxygenase by baicalein induces microglia PPAR β/δ: a potential therapeutic role for CNS autoimmune disease. Cell Death Dis. (2013) 4:e569. 10.1038/cddis.2013.86 23559003 PMC3668632

[74] Fakan B Szalardy L Vecsei L. Exploiting the Therapeutic Potential of Endogenous Immunomodulatory Systems in Multiple Sclerosis-Special Focus on the Peroxisome Proliferator-Activated Receptors (PPARs) and the Kynurenines. Int J Mol Sci. (2019) 20:426. 10.3390/ijms20020426 30669473 PMC6358998

[75] Ying S Yang H Gu Q Wu Z Zou N Wang C-Z. The small-molecule compound baicalein alleviates experimental autoimmune encephalomyelitis by suppressing pathogenetic CXCR6^+^ Cd4 cells. Int immunopharmacol. (2023) 114:109562. 10.1016/j.intimp.2022.109562 36508914

[76] Ma X Wang S Li C Jia X Wang T Leng Z. Baicalein inhibits the polarization of microglia/macrophages to the M1 phenotype by targeting STAT1 in EAE mice. Int Immunopharmacol. (2022) 113:109373. 10.1016/j.intimp.2022.109373 36279665

[77] Tamilselvam K Nataraj J Janakiraman U Manivasagam T Essa M. Antioxidant and anti-inflammatory potential of hesperidin against 1-methyl-4-phenyl-1, 2, 3, 6-tetrahydropyridine-induced experimental Parkinson's disease in mice. Int J Nutr Pharmacol. (2013) 3:294–302. 10.4103/2231-0738.114875

[78] Haghmorad D Mahmoudi MB Salehipour Z Jalayer Z Brojeni AAM Rastin M. Hesperidin ameliorates immunological outcome and reduces neuroinflammation in the mouse model of multiple sclerosis. J Neuroimmunol. (2017) 302:23–33. 10.1016/j.jneuroim.2016.11.009 27912911

[79] Sun C-P Zhou J-J Yu Z-L Huo X-K Zhang J Morisseau C. Kurarinone alleviated Parkinson's disease *via* stabilization of epoxyeicosatrienoic acids in animal model. Proc Natl Acad Sci U S A. (2022) 119:e2118818119. 10.1073/pnas.2118818119 35217618 PMC8892522

[80] Xie L Gong W Chen J Xie H-w Wang M Yin X-p. The flavonoid kurarinone inhibits clinical progression of EAE through inhibiting Th1 and Th17 cell differentiation and proliferation. Int Immunopharmacol. (2018) 62:227–36. 10.1016/j.intimp.2018.06.022 30031314

[81] Zhao W Ma L Cai C Gong X. Caffeine inhibits NLRP3 inflammasome activation by suppressing MAPK/NF-κP and A2aR signaling in LPS-induced THP-1 macrophages. Int J Biol Sci. (2019) 15:1571–81. 10.7150/ijbs.34211 31360100 PMC6643212

[82] Basu Mallik S Mudgal J Hall S Kinra M Grant GD Nampoothiri M. Remedial effects of caffeine against depressive-like behaviour in mice by modulation of neuroinflammation and BDNF. Nutr Neurosci. (2022) 25:1836–44. 10.1080/1028415X.2021.1906393 33814004

[83] Chen GQ Chen YY Wang XS Wu SZ Yang HM Xu HQ. Chronic caffeine treatment attenuates experimental autoimmune encephalomyelitis induced by guinea pig spinal cord homogenates in wistar rats. Brain Res. (2010) 1309:116–25. 10.1016/j.brainres.2009.10.054 19879252

[84] Shrivastava P Vaibhav K Tabassum R Khan A Ishrat T Khan MM. Anti-apoptotic and anti-inflammatory effect of piperine on 6-OHDA induced Parkinson's rat model. J Nutr Biochem. (2013) 24:680–7. 10.1016/j.jnutbio.2012.03.018 22819561

[85] Nasrnezhad R Halalkhor S Sadeghi F Pourabdolhossein F. Piperine improves experimental autoimmune encephalomyelitis (EAE) in lewis rats through its neuroprotective, anti-inflammatory, and antioxidant effects. Mol Neurobiol. (2021) 58:5473–93. 10.1007/s12035-021-02497-5 34338970

[86] Ojha S Javed H Azimullah S Khair SBA Haque ME. Glycyrrhizic acid attenuates neuroinflammation and oxidative stress in rotenone model of Parkinson's disease. Neurotox Res. (2016) 29:275–87. 10.1007/s12640-015-9579-z 26607911

[87] Santoro M Maetzler W Stathakos P Martin HL Hobert MA Rattay TW. *In-vivo* evidence that high mobility group box 1 exerts deleterious effects in the 1-methyl-4-phenyl-1,2,3,6-tetrahydropyridine model and Parkinson's disease which can be attenuated by glycyrrhizin. Neurobiol Dis. (2016) 91:59–68. 10.1016/j.nbd.2016.02.018 26921471 PMC4867789

[88] Sun Y Chen H Dai J Wan Z Xiong P Xu Y. Glycyrrhizin protects mice against experimental autoimmune encephalomyelitis by inhibiting high-mobility group box 1 (HMGB1) expression and neuronal Hmgb1 release. Front Immunol. (2018) 9:1518. 10.3389/fimmu.2018.01518 30013568 PMC6036111

[89] Zhang L Previn R Lu L Liao R-F Jin Y Wang R-K. Crocin, a natural product attenuates lipopolysaccharide-induced anxiety and depressive-like behaviors through suppressing NF-κB and NLRP3 signaling pathway. Brain Res Bull. (2018) 142:352–9. 10.1016/j.brainresbull.2018.08.021 30179677

[90] Deslauriers AM Afkhami-Goli A Paul AM Bhat RK Acharjee S Ellestad KK. Neuroinflammation and endoplasmic reticulum stress are coregulated by crocin to prevent demyelination and neurodegeneration. J Immunol. (2011) 187:4788–99. 10.4049/jimmunol.1004111 21964030

[91] Ghiasian M Khamisabadi F Kheiripour N Karami M Haddadi R Ghaleiha A. Effects of crocin in reducing DNA damage, inflammation, and oxidative stress in multiple sclerosis patients: a double-blind, randomized, and placebo-controlled trial. J Biochem Mol Toxicol. (2019) 33:e22410. 10.1002/jbt.22410 31617649

[92] Chen Q Lei Y-Q Liu J-F Wang Z-C Cao H. Beneficial effects of chlorogenic acid treatment on neuroinflammation after deep hypothermic circulatory arrest may be mediated through CYLD/NF-Kb Signaling. Brain Res. (2021) 1767:147572. 10.1016/j.brainres.2021.147572 34216581

[93] Singh SS Rai SN Birla H Zahra W Kumar G Gedda MR. Effect of chlorogenic acid supplementation in MPTP-intoxicated mouse. Front Pharmacol. (2018) 9:757. 10.3389/fphar.2018.00757 30127737 PMC6087758

[94] Wróbel-Biedrawa D Grabowska K Galanty A Sobolewska D Podolak I. A flavonoid on the brain: quercetin as a potential therapeutic agent in central nervous system disorders. Life. (2022) 12:591. 10.3390/life12040591 35455082 PMC9027262

[95] Chetia Phukan B Dutta A Deb S Saikia R Mazumder MK Paul R. Garcinol blocks motor behavioural deficits by providing dopaminergic neuroprotection in MPTP mouse model of Parkinson's disease: involvement of anti-inflammatory response. Exp Brain Res. (2022) 240:113–22. 10.1007/s00221-021-06237-y 34633467

[96] Yu W-B Wang Q Chen S Cao L Tang J Ma C-G. The therapeutic potential of ginkgolide K in experimental autoimmune encephalomyelitis *via* peripheral immunomodulation. Int Immunopharmacol. (2019) 70:284–94. 10.1016/j.intimp.2019.02.035 30851709

[97] Li Q-Y Miao Q Sui R-X Cao L Ma C-G Xiao B-G. Ginkgolide K supports remyelination via induction of astrocytic IGF/PI3K/Nrf2 axis. Int Immunopharmacol. (2019) 75:105819. 10.1016/j.intimp.2019.105819 31421546

[98] Chen S Zhang J Yu W-B Zhuang J-C Xiao W Wu Z-Y. Eomesodermin in Cd4^+^ T cells is essential for ginkgolide K ameliorating disease progression in experimental autoimmune encephalomyelitis. Int J Biol Sci. (2021) 17:50–61. 10.7150/ijbs.50041 33390832 PMC7757039

[99] Gutierrez B Gallardo I Ruiz L Alvarez Y Cachofeiro V Margolles A. Oleanolic acid ameliorates intestinal alterations associated with eae. J Neuroinflammation. (2020) 17:363. 10.1186/s12974-020-02042-6 33246492 PMC7697371

[100] Tseng H-C Wang M-H Chang K-C Soung H-S Fang C-H Lin Y-W. Protective effect of (-)epigallocatechin-3-gallate on rotenone-induced parkinsonism-like symptoms in rats. Neurotox Res. (2020) 37:669–82. 10.1007/s12640-019-00143-6 31811588

[101] Martinez-Perez DA Jimenez-Del-Rio M Velez-Pardo C. Epigallocatechin-3-gallate protects and prevents paraquat-induced oxidative stress and neurodegeneration in knockdown Dj-1-Beta drosophila melanogaster. Neurotox Res. (2018) 34:401–16. 10.1007/s12640-018-9899-x 29667128

[102] Gaballah HH Zakaria SS Elbatsh MM Tahoon NM. Modulatory effects of resveratrol on endoplasmic reticulum stress-associated apoptosis and oxido-inflammatory markers in a rat model of rotenone-induced Parkinson's disease. Chem Biol Interact. (2016) 251:10–6. 10.1016/j.cbi.2016.03.023 27016191

[103] Li H Shen Y Xiao H Sun W. Resveratrol attenuates rotenone-induced inflammation and oxidative stress *via* Stat1 and Nrf2/Keap1/SLC7A11 pathway in a microglia cell line. Pathol Res Pract. (2021) 225:153576. 10.1016/j.prp.2021.153576 34391968

[104] Lin X Bai D Wei Z Zhang Y Huang Y Deng H. Curcumin attenuates oxidative stress in RAW2647 cells by increasing the activity of antioxidant enzymes and activating the Nrf2-Keap1 pathway. PLoS ONE. (2019) 14:e0216711. 10.1371/journal.pone.0216711 31112588 PMC6528975

[105] Jaroonwitchawan T Chaicharoenaudomrung N Natnkaew J Noisa P. Curcumin attenuates paraquat-induced cell death in human neuroblastoma cells through modulating oxidative stress and autophagy. Neurosci Lett. (2017) 636:40–7. 10.1016/j.neulet.2016.10.050 27793699

[106] Khodaei F Khoshnoud MJ Heidaryfar S Heidari R Karimpour Baseri MH Azarpira N. The effect of ellagic acid on spinal cord and sciatica function in a mice model of multiple sclerosis. J Biochem Mol Toxicol. (2020) 34:e22564. 10.1002/jbt.22564 32640490

[107] Zhang Z Cui W Li G Yuan S Xu D Hoi MP. Baicalein protects against 6-OHDA-induced neurotoxicity through activation of Keap1/Nrf2/HO-1 and involving PKCα and PI3K/Akt signaling pathways. J Agric Food Chem. (2012) 60:8171–82. 10.1021/jf301511m 22838648

[108] Parhiz H Roohbakhsh A Soltani F Rezaee R Iranshahi M. Antioxidant and anti-inflammatory properties of the citrus flavonoids hesperidin and hesperetin: an updated review of their molecular mechanisms and experimental models. Phytother Res. (2015) 29:323–31. 10.1002/ptr.5256 25394264

[109] Bayazid AB Lim BO. Quercetin is an active agent in berries against neurodegenerative diseases progression through modulation of Nrf2/HO1. Nutrients. (2022) 14:5132. 10.3390/nu14235132 36501161 PMC9737775

[110] Badshah H Ikram M Ali W Ahmad S Hahm JR Kim MO. Caffeine may abrogate LPS-induced oxidative stress and neuroinflammation by regulating Nrf2/TLR4 in adult mouse brains. Biomolecules. (2019) 9:719. 10.3390/biom9110719 31717470 PMC6921022

[111] Garabadu D Agrawal N. Naringin exhibits neuroprotection against rotenone-induced neurotoxicity in experimental rodents. Neuromolecular Med. (2020) 22:314–30. 10.1007/s12017-019-08590-2 31916219

[112] Haeri P Mohammadipour A Heidari Z Ebrahimzadeh-bideskan A. Neuroprotective effect of crocin on substantia Nigra in MPTP-induced Parkinson's disease model of mice. Anat Sci Int. (2019) 94:119–27. 10.1007/s12565-018-0457-7 30159851

[113] Zhang Z Peng L Fu Y Wang W Wang P Zhou F. Ginnalin a binds to the subpockets of Keap1 kelch domain to activate the Nrf2-regulated antioxidant defense system in SH-SY5Y cells. ACS Chem Neurosci. (2021) 12:872–82. 10.1021/acschemneuro.0c00713 33571414

[114] Engelbrecht I Petzer JP Petzer A. Evaluation of selected natural compounds as dual inhibitors of catechol-*O*-methyltransferase and monoamine oxidase. Cent Nerv Syst Agents Med Chem. (2019) 19:133–45. 10.2174/1871524919666190619090852 31258092

[115] Petzer A Pienaar A Petzer JP. The interactions of caffeine with monoamine oxidase. Life Sci. (2013) 93:283–7. 10.1016/j.lfs.2013.06.020 23850513

[116] Parrales-Macias V Harfouche A Ferrie L Haik S Michel PP Raisman-Vozari R. Effects of a new natural catechol-*O*-methyl transferase inhibitor on two *in vivo* models of Parkinson's disease. ACS Chem Neurosci. (2022) 12:3303–13. 10.1021/acschemneuro.2c00356 36347018

[117] Sharma N Nehru B. Curcumin affords neuroprotection and inhibits α-synuclein aggregation in lipopolysaccharide-induced Parkinson's disease model. Inflammopharmacology. (2018) 26:349–60. 10.1007/s10787-017-0402-8 29027056

[118] Zhu M Rajamani S Kaylor J Han S Zhou F Fink AL. The flavonoid baicalein inhibits fibrillation of α-synuclein and disaggregates existing fibrils. J Biol Chem. (2004) 279:26846–57. 10.1074/jbc.M403129200 15096521

[119] Yao Y Tang Y Zhou Y Yang Z Wei G. Baicalein exhibits differential effects and mechanisms towards disruption of α-synuclein fibrils with different polymorphs. Int J Biol Macromol. (2022) 220:316–25. 10.1016/j.ijbiomac.2022.08.088 35981677

[120] Saffari B Amininasab M. Crocin inhibits the fibrillation of human α-synuclein and disassembles mature fibrils: experimental findings and mechanistic insights from molecular dynamics simulation. ACS Chem Neurosci. (2021) 12:4037–57. 10.1021/acschemneuro.1c00379 34636232

[121] Fan Q Liu Y Wang X Zhang Z Fu Y Liu L. Ginnalin A inhibits aggregation, reverses fibrillogenesis, and alleviates cytotoxicity of amyloid β(1-42). ACS Chem Neurosci. (2020) 11:638–47. 10.1021/acschemneuro.9b00673 31967782

[122] Ghanem SS Fayed HS Zhu Q Lu J-H Vaikath NN Ponraj J. Natural alkaloid compounds as inhibitors for α-synuclein seeded fibril formation and toxicity. Molecules. (2021) 26:3736. 10.3390/molecules26123736 34205249 PMC8234408

[123] Perni M Galvagnion C Maltsev A Meisl G Mueller MBD Challa PK. A natural product inhibits the initiation of α-synuclein aggregation and suppresses its toxicity. Proc Natl Acad Sci U S A. (2017) 114:E1009–E17. 10.1073/pnas.1701964114 28096355 PMC5307473

[124] Perni M Flagmeier P Limbocker R Cascella R Aprile FA Galvagnion C. Multistep inhibition of α-synuclein aggregation and toxicity *in vitro* and *in vivo* by trodusquemine. ACS Chem Biol. (2018) 13:2308–19. 10.1021/acschembio.8b00466 29953201

[125] West CL Mao Y-K Delungahawatta T Amin JY Farhin S McQuade RM. Squalamine restores the function of the enteric nervous system in mouse models of Parkinson's disease. J Parkinsons Dis. (2020) 10:1477–91. 10.3233/JPD-202076 32925094

[126] Luan Y Ren X Zheng W Zeng Z Guo Y Hou Z. Chronic caffeine treatment protects against α-synucleinopathy by reestablishing autophagy activity in the mouse striatum. Front Neurosci. (2018) 12:301. 10.3389/fnins.2018.00301 29770111 PMC5942142

[127] Bang Y Kwon Y Kim M Moon SH Jung K Choi HJ. Ursolic acid enhances autophagic clearance and ameliorates motor and non-motor symptoms in Parkinson's disease mice model. Acta Pharmacol Sin. (2022). 10.1038/s41401-022-00988-2. [Epub ahead of print]. 36138143 PMC10042858

[128] Yang G Li J Cai Y Yang Z Li R Fu W. Glycyrrhizic acid alleviates 6-hydroxydopamine and corticosterone-induced neurotoxicity in SH-SY5Y cells through modulating autophagy. Neurochem Res. (2018) 43:1914–26. 10.1007/s11064-018-2609-5 30206804

[129] Rusmini P Cortese K Crippa V Cristofani R Cicardi ME Ferrari V. Trehalose induces autophagy *via* lysosomal-mediated TFEB activation in models of motoneuron degeneration. Autophagy. (2019) 15:631–51. 10.1080/15548627.2018.1535292 30335591 PMC6526812

[130] Leri M Vasarri M Palazzi L Barletta E Nielsen E Bucciantini M. Maysin plays a protective role against α-synuclein oligomers cytotoxicity by triggering autophagy activation. Food Chem Toxicol. (2020) 144:111626. 10.1016/j.fct.2020.111626 32738375

[131] Busto R Serna J Perianes-Cachero A Quintana-Portillo R Garcia-Seisdedos D Canfran-Duque A. Ellagic acid protects from myelin-associated sphingolipid loss in experimental autoimmune encephalomyelitis. Biochim Biophys Acta Mol Cell Biol Lipids. (2018) 1863:958–67. 10.1016/j.bbalip.2018.05.009 29793057

[132] Roshanbakhsh H Salmani ME Dehghan S Nazari A Javan M Pourabdolhossein F. Piperine ameliorated memory impairment and myelin damage in lysolecethin induced hippocampal demyelination. Life Sci. (2020) 253:117671. 10.1016/j.lfs.2020.117671 32335165

[133] Zhang Y Li X Ciric B Curtis MT Chen W-J Rostami A. A dual effect of ursolic acid to the treatment of multiple sclerosis through both immunomodulation and direct remyelination. Proc Natl Acad Sci U S A. (2020) 117:9082–93. 10.1073/pnas.2000208117 32253301 PMC7183235

[134] Honarvar F Hojati V Zare L Bakhtiari N Javan M. Ursolic acid enhances myelin repair in adult mice brains and stimulates exhausted oligodendrocyte progenitors to remyelinate. J Mol Neurosci. (2022) 72:2081–93. 10.1007/s12031-022-02059-x 35976486

[135] Yamamoto S Sakemoto C Iwasa K Maruyama K Shimizu K Yoshikawa K. Ursolic acid treatment suppresses cuprizone-induced demyelination and motor dysfunction *via* upregulation of Igf-1. J Pharmacol Sci. (2020) 144:119–22. 10.1016/j.jphs.2020.08.002 32921392

[136] Wang D Li S-P Fu J-S Zhang S Bai L Guo L. Resveratrol defends blood-brain barrier integrity in experimental autoimmune encephalomyelitis mice. J Neurophysiol. (2016) 116:2173–9. 10.1152/jn.00510.2016 27535376 PMC5102308

[137] Niu X Sang H Wang J. Naringenin attenuates experimental autoimmune encephalomyelitis by protecting the intact of blood-brain barrier and controlling inflammatory cell migration. J Nutr Biochem. (2021) 89:108560. 10.1016/j.jnutbio.2020.108560 33249188

[138] Lee MJ Choi JH Oh J Lee YH In J-G Chang B-J. Rg3-enriched Korean red ginseng extract inhibits blood-brain barrier disruption in an animal model of multiple sclerosis by modulating expression of Nadph oxidase 2 and 4. J Ginseng Res. (2021) 45:433–41. 10.1016/j.jgr.2020.09.001 34025136 PMC8134843

[139] Jing Y Ma R Chu Y Dou M Wang M Li X. Matrine treatment induced an A2 astrocyte phenotype and protected the blood-brain barrier in CNS autoimmunity. J Chem Neuroanat. (2021) 117:102004. 10.1016/j.jchemneu.2021.102004 34280490

