# Corrigendum: Natural products targeting cellular processes common in Parkinson's disease and multiple sclerosis

**DOI:** 10.3389/fneur.2023.1350223

**Published:** 2023-12-22

**Authors:** Xuxu Xu, Chaowei Han, Pengcheng Wang, Feimeng Zhou

**Affiliations:** ^1^Institute of Surface Analysis and Chemical Biology, The University of Jinan, Jinan, Shangdong, China; ^2^Department of Neurology, Shandong Key Laboratory of Rheumatic Disease and Translational Medicine, The First Affiliated Hospital of Shandong First Medical University and Shandong Provincial Qianfoshan Hospital, Shandong Institute of Neuroimmunology, Jinan, Shandong, China

**Keywords:** Parkinson's disease, multiple sclerosis, natural products, neuroinflammation, oxidative stress, cellular process

In the published article, there was an error in the order of Affiliations. Instead of “^1^Department of Neurology, Shandong Key Laboratory of Rheumatic Disease and Translational Medicine, The First Affiliated Hospital of Shandong First Medical University and Shandong Provincial Qianfoshan Hospital, Shandong Institute of Neuroimmunology, Jinan, Shandong, China. ^2^Institute of Surface Analysis and Chemical Biology, The University of Jinan, Jinan, Shangdong, China”, it should be “^1^Institute of Surface Analysis and Chemical Biology, The University of Jinan, Jinan, Shangdong, China. ^2^Department of Neurology, Shandong Key Laboratory of Rheumatic Disease and Translational Medicine, The First Affiliated Hospital of Shandong First Medical University and Shandong Provincial Qianfoshan Hospital, Shandong Institute of Neuroimmunology, Jinan, Shandong, China”.

In the published article, there was an error in the Funding statement. The Funding number was incorrect. The statement was incorrectly written as “The authors would like to thank the financial support from the Natural Science Foundation of China (Nos. 21906065 and 82001825), Natural Science Foundation of Shandong Province (ZR2020QH113), and the Shandong Provincial Grant for the Talent-Leading Teams.” The correct Funding statement appears below.

The authors would like to thank the financial support from the Natural Science Foundation of China (Nos. 21906065 and 82001285), Natural Science Foundation of Shandong Province (ZR2020QH113) and the Shandong Provincial Grant for the Talent-Leading Teams.

The authors apologize for these errors and state that this does not change the scientific conclusions of the article in any way. The original article has been updated.

